# Factors Associated with Non-attendance at a Follow-up Visit for Dyslipidemia Identified at Health Checkups: A Retrospective Cohort Study in a Japanese Prefecture

**DOI:** 10.31662/jmaj.2024-0065

**Published:** 2024-10-03

**Authors:** Yuta Taniguchi, Masao Iwagami, Takehiro Sugiyama, Naoaki Kuroda, Takuya Yamaoka, Ryota Inokuchi, Ai Suzuki, Taeko Watanabe, Fujiko Irie, Nanako Tamiya

**Affiliations:** 1 Department of Health Services Research, Graduate School of Comprehensive Human Sciences, University of Tsukuba, Tsukuba, Japan; 2 Institute for Global Health Policy Research, Bureau of International Health Cooperation, National Center for Global Health and Medicine, Tokyo, Japan; 3 Department of Health Services Research, Institute of Medicine, University of Tsukuba, Tsukuba, Japan; 4 Health Services Research and Development Center, University of Tsukuba, Tsukuba, Japan; 5 Faculty of Epidemiology and Population Health, London School of Hygiene and Tropical Medicine, London, United Kingdom; 6 Digital Society Division, Cyber Medicine Research Center, University of Tsukuba, Tsukuba, Japan; 7 Diabetes and Metabolism Information Center, Research Institute, National Center for Global Health and Medicine, Tokyo, Japan; 8 Department of Public Mental Health Research, National Institute of Mental Health, National Center for Neurology and Psychiatry, Tokyo, Japan; 9 Health Department, Tsukuba City, Tsukuba, Japan; 10 Department of Emergency and Critical Care Medicine, The University of Tokyo Hospital, Tokyo, Japan; 11 Tsuchiura Public Health Center of Ibaraki Prefectural Government, Tsuchiura, Japan

**Keywords:** Health checkup, Dyslipidemia, Follow-up visit, Japan

## Abstract

**Introduction:**

Dyslipidemia increases the risk of cardiovascular and cerebrovascular diseases. Visiting a physician for follow-up is essential when dyslipidemia is detected during health checkups. We investigated factors associated with non-attendance at a follow-up visit for dyslipidemia.

**Methods:**

We conducted a retrospective cohort study using linked health checkups and medical claims data from individuals covered by National Health Insurance in Ibaraki Prefecture, Japan. Participants were 40-74 years old, underwent health checkups between April 2018 and March 2019, and had cholesterol levels exceeding the recommended levels to visit a physician. We excluded individuals who had visited physicians for dyslipidemia in the past year. We calculated the proportion of patients who had a follow-up visit with a physician within 180 days after their health checkup. Then, we investigated the demographic and clinical characteristics associated with non-attendance using a multivariable logistic regression model.

**Results:**

Among 33,503 individuals (median age, 66 years [interquartile range, 59-69 years]; females, 58.8%) with dyslipidemia at the health checkup, 18.1% attended follow-up visits. Younger age, male sex, drinking habits, and lack of symptoms were associated with higher odds of non-attendance. Participants who underwent health checkups at public facilities, lacked other abnormal results at health checkups, and had not visited physicians for other diseases were less likely to attend a follow-up visit. Among those with elevated low-density lipoprotein cholesterol (LDL-C) levels, those with relatively lower LDL-C levels were less likely to attend.

**Conclusions:**

Systems that inform high-risk populations of non-attendance and encourage follow-up visits are warranted.

## Introduction

Dyslipidemia is a risk factor for cardiovascular disease and ischemic stroke ^(1), (2), (3)^. The global burden of dyslipidemia has been increasing ^(4)^, and 4.5 million deaths were reportedly attributable to high low-density lipoprotein cholesterol (LDL-C) in 2020, with a population attributable fraction of 8.0% ^(5)^.

The estimated number of cases of dyslipidemia in Japan was 2.2 million in 2017 ^(6)^. To reduce noncommunicable diseases, such as cardiovascular disease and diabetes, the Japanese government introduced a specific health checkup system in 2008. It obliged all insurers to provide annual health checkups for insured persons aged 40-74 years ^(7)^. When abnormal test results are detected at the health checkup, the recipients are advised to visit a physician for follow-up, often via report comments. Nevertheless, several studies suggested that most individuals with abnormal test results do not attend a follow-up physician visit within 6 months of the checkup: only 15%-21% for dyslipidemia ^(8)^, 21%-49% for hyperglycemia ^(9), (10), (11)^, and 11% for high blood pressure ^(12)^. A study suggested that fewer physician visits before the health checkup, lower HbA1c levels, and a lack of history of treatment for dyslipidemia or hypertension were predictive factors for non-attendance at hyperglycemia follow-ups ^(10)^.

However, the factors associated with non-attendance at follow-up visits for dyslipidemia remain unknown. Early intervention in dyslipidemia, such as lifestyle modification and lipid-lowering medications, is crucial to preventing cardiovascular disease ^(13), (14)^. Thus, identifying the population at high risk of non-attendance at follow-ups is essential for healthcare providers and policymakers. This study aimed to investigate factors associated with non-attendance at a follow-up physician visit after dyslipidemia had been identified at health checkups.

## Materials and Methods

### Data source

We obtained anonymously linked data from specific health checkups and medical claims of the National Health Insurance in Ibaraki Prefecture, Japan. The National Health Insurance in Japan is a municipality-based insurance that covers individuals aged < 75 years who are self-employed, unemployed, or working part-time ^(7)^. The database included all insured individuals. The insurers (i.e., municipalities) administered the original data and anonymized it before providing it to the researchers.

In 2018, there were approximately 735,000 insured persons in Ibaraki Prefecture, constituting one-fourth of the total population ^(15)^, with 42% aged 65 or older ^(16)^. In Japan, insurers are legally required to provide specific health checkups annually to insured persons aged 40-74 years. However, insured persons face no penalties for not attending the health checkup, with only 38% attending in 2018 in Ibaraki Prefecture ^(17)^.

The University of Tsukuba Ethics Committee (approval no. 1845-1) approved this study per the Declaration of Helsinki. Individual participants’ consent was waived because the data were anonymized before the researchers received them.

### Study population

We included individuals who received specific health checkups between April 2018 and March 2019 and whose cholesterol levels exceeded at least one of the recommended levels to visit a physician: LDL-C ≥ 140 mg/dL, triglycerides (TG) ≥ 300 mg/dL, or high-density lipoprotein cholesterol (HDL-C) ≤ 34 mg/dL ^(18)^.

We excluded individuals with records of previous physician visits for dyslipidemia within 1 year before the health checkup. A physician visit for dyslipidemia was defined as either a recorded (determined or possible) diagnosis of dyslipidemia (International Statistical Classification of Diseases and Related Health Problems 10th Revision [ICD-10] code of E78), a blood test for cholesterol, or a prescription for dyslipidemia medication (World Health Organization [WHO] Anatomical Therapeutic Chemical [ATC] code of C10). We also excluded individuals who entered the National Health Insurance in May 2017 or later (to obtain information on the exposure variables) and those who left within 180 days after the health checkup (to get information on the outcome). In addition, individuals with missing values of exposure and outcome variables and those with implausible body mass index (BMI) values (<14 or ≥40 kg/m^2^) were excluded ^(19)^.

### Outcome

The outcome was non-attendance at a follow-up physician visit for dyslipidemia within 180 days after the health checkup, in accordance with previous reports ^(9), (10), (11), (12)^. To define a physician visit for dyslipidemia, we used the same criteria mentioned earlier: a recorded diagnosis of dyslipidemia, a blood test for cholesterol, or a prescription for a dyslipidemia medication.

### Exposures for non-attendance

**Figure 1.**
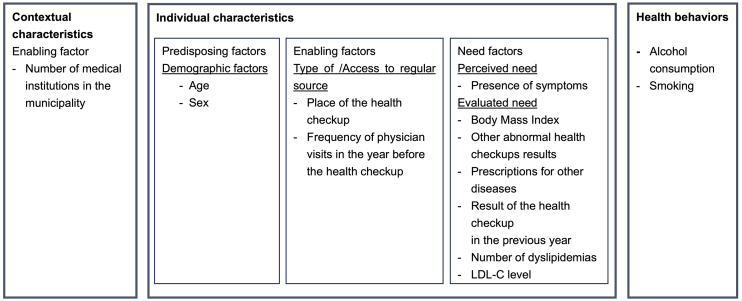
Exposure variables based on Andersen’s behavioral model. LDL-C, low-density lipoprotein cholesterol.

Based on previous studies ^(9), (10), (11), (12)^ and the authors’ expertise, we selected exposure variables according to the theoretical framework of Andersen’s behavioral model of health services use (classified into predisposing, enabling, and need factors), as shown in Figure 1 ^(20)^. Briefly, predisposing factors include demographic and socio-structural characteristics and health beliefs. Enabling factors refer to the conditions that make services available to individuals, including regular sources of care and accessibility of the source. Need factors consist of perceived and evaluated illness ^(20)^.

As predisposing factors, we included age and sex. As enabling factors, we included the place of health checkups and the frequency of physician visits in the year before the health checkups. The place of health checkups was dichotomized into public facilities (e.g., community health centers and city halls) or medical institutions. In general, individuals can choose whether they receive health checkups at a public facility (also called “*shudan kenshin*”) or a medical institution (“*iryokikan kenshin*”), and both are free of charge. The frequency of physician visits in the year before the health checkup was a continuous variable, defined as the number of months the participants received medical services in the year before the health checkup. In addition, as a contextual (i.e., regional) characteristic, we included the number of medical institutions (hospitals and clinics) per 1,000 population in the participants’ municipalities based on the Survey of Medical Institutions conducted by the Ministry of Health, Labour and Welfare in 2018 ^(21)^.

As need factors, we included variables representing the perceived and evaluated needs. For perceived need, we included a binary variable on the presence of any subjective symptom (yes/no) in the questionnaire during the health checkup. For evaluated need, based on the health checkup test results, we included a categorical variable for BMI (<25, 25-30, or ≥30 kg/m^2^) and binary variables for hypertension (systolic blood pressure ≥ 140 mmHg or diastolic blood pressure ≥ 90 mmHg), hyperglycemia (fasting blood sugar ≥ 126 mg/dL or HbA1c ≥ 6.5%), liver dysfunction (aspartate aminotransferase [AST] > 50 U/L, alanine aminotransferase [ALT] > 50 U/L, or γ-glutamyl transpeptidase [γ-GTP] > 100 U/L), and urinary dipstick results (proteinuria and glucosuria). We also included the number of dyslipidemias identified at the health checkup (high LDL-C, low HDL-C, or high TG), which took the value of one, two, or three. We included binary variables from the medical claims data indicating prior prescriptions for hypertension (WHO ATC codes: C02, C03, C04, C07, C08, or C09), diabetes (A10A or A10B), hyperuricemia (M04), and depression (N06A or N06BA) within 1 year before the health checkups. Lastly, the health checkup result from the previous fiscal year (April 2017 to March 2018) was included as a categorical variable: dyslipidemia, no dyslipidemia, or no health checkups.

As health behaviors, we included alcohol consumption frequency (daily, occasionally, or rarely) and current smoking status (yes/no) as categorical variables.

### Statistical analysis

First, we calculated the proportion of patients who attended a follow-up physician visit within 180 days after dyslipidemia was identified at the health checkup, overall and by type, combination, and degree of dyslipidemia. We also calculated the proportion in each municipality in Ibaraki Prefecture (n = 44) and stratified by the municipality population size (urban area; ≥30,000; rural area; <30,000). Additionally, we calculated the proportion of patients who attended a follow-up physician visit within 365 days. Second, we conducted univariable and multivariable logistic regression analyses to identify factors significantly associated with non-attendance at a follow-up visit. In addition to the exposure variables described above, we used clustered standard errors at the municipal level to account for potential correlations among participants in the same municipality. We evaluated the multicollinearity between exposure variables using the Pearson correlation coefficient, smaller than 0.5, between any two exposure variables; therefore, we included all the variables in the multivariable logistic regression model. Supplementary Table 1 presents the profiles of 44 municipalities in Ibaraki Prefecture.

As an additional analysis, we restricted the analyses to individuals with high LDL-C levels by excluding those with high TG or low HDL-C levels only. For this analysis, we assessed the association between LDL-C levels and the outcome using the following two methods. First, to evaluate the possibility of a nonlinear relationship, we included a categorical variable of LDL-C level (140-160 mg/dL, 160-180 mg/dL, or higher) in the multivariable logistic regression. Second, we applied the restricted cubic spline function with five knots at the 5, 27.5, 50, 72.5, and 95th percentiles of the LDL-C level ^(22)^.

Two-sided P-values < 0.05 were considered statistically significant. All analyses were performed using the Stata 17 software (StataCorp, College Station, TX, USA).

## Results

**Figure 2.**
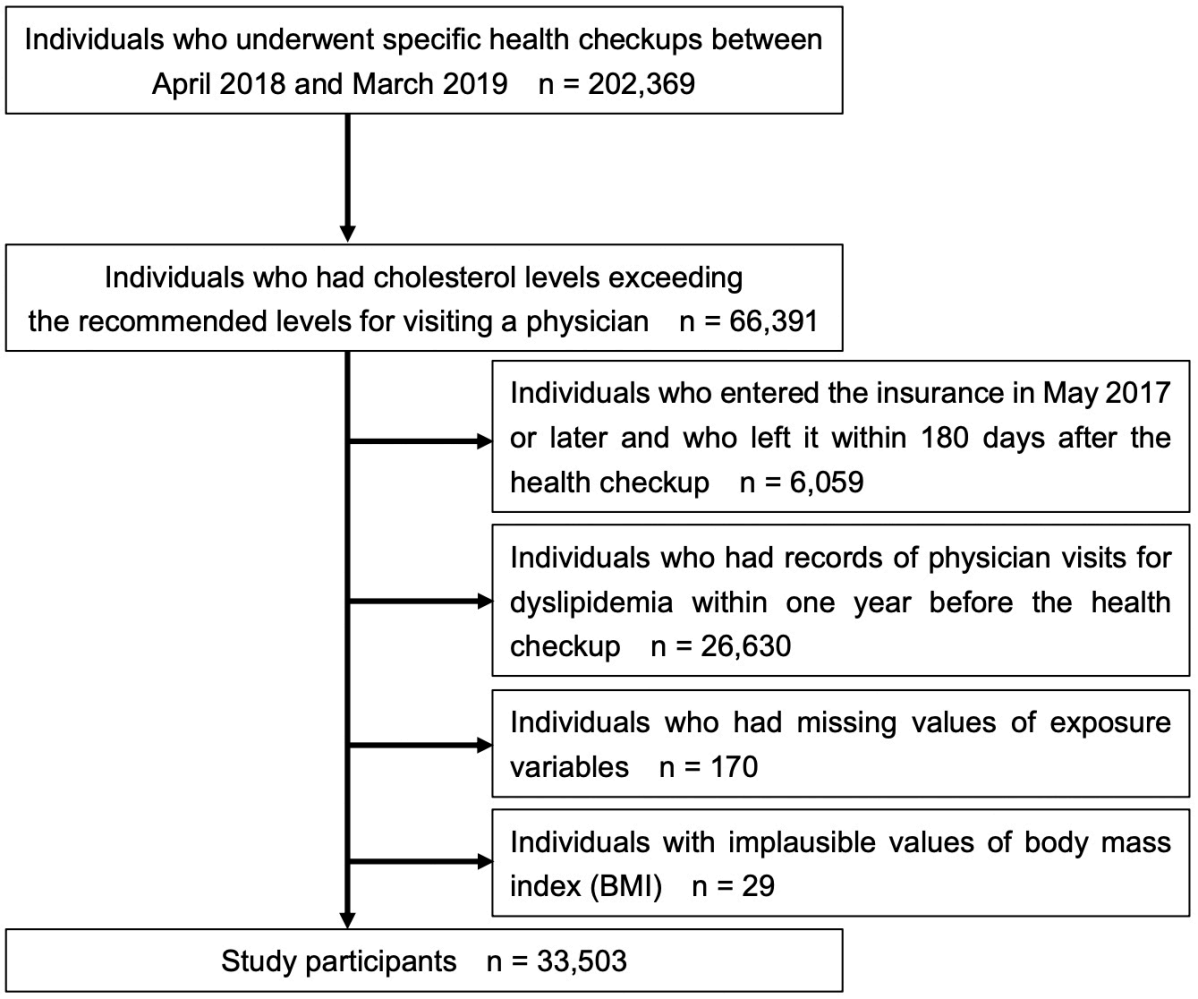
Flowchart of the participant selection.

Figure 2 shows a participant selection flowchart. Among the 202,369 individuals who underwent specific health checkups between April 2018 and March 2019, 66,391 had cholesterol levels exceeding the recommended levels for visiting a physician. After excluding 26,630 individuals with records of physician visits for dyslipidemia within 1 year before the health checkup, we included 33,503 individuals in the analysis. The median age was 66 years (interquartile range [IQR], 59-69), and 58.8% were female (Table 1). Regarding the type of dyslipidemia, 31,084 (92.8%) individuals had high LDL-C, 2,779 (8.3%) had high TG, and 938 (2.8%) had low HDL-C levels. Their median (IQR) cholesterol values were 158 (148-173) mg/dL for LDL-C in those with high LDL-C, 368 (327-450) mg/dL for TG in those with high TG, and 32 (31-34) mg/dL for HDL-C in those with low HDL-C.

**Table 1.** Study Participant Characteristics.

	Attended a follow-up visit	Did not attend a follow-up visit	Total
	(n = 6,052)	(n = 27,451)	(n = 33,503)
Age, median (IQR), year	67 (61-70)	66 (58-69)	66 (59-69)
Sex (female)	3,805 (62.9)	15,909 (58.0)	19,714 (58.8)
BMI, kg/m^2^
<25	4,362 (72.1)	20,062 (73.1)	24,424 (72.9)
25-30	1,465 (24.2)	6,482 (23.6)	7,947 (23.7)
≥30	225 (3.7)	907 (3.3)	1,132 (3.4)
Current smoker, yes	810 (13.4)	4,271 (15.6)	5,081 (15.2)
Frequency of alcohol consumption
Every day	1,140 (18.8)	5,704 (20.8)	6,844 (20.4)
Occasionally	1,180 (19.5)	5,863 (21.4)	7,043 (21.0)
Rarely	3,732 (61.7)	15,884 (57.9)	19,616 (58.6)
Symptoms, yes	1,939 (32.0)	7,458 (27.2)	9,397 (28.1)
Health checkup results
Hypertension	1,738 (28.7)	7,022 (25.6)	8,760 (26.2)
Hyperglycemia	619 (10.2)	1,427 (5.2)	2,046 (6.1)
Liver dysfunction	571 (9.4)	1,984 (7.2)	2,555 (7.6)
Proteinuria	227 (3.8)	501 (1.8)	728 (2.2)
Glycosuria	200 (3.3)	463 (1.7)	663 (2.0)
Number of dyslipidemias at the health checkup
1	5,798 (95.7)	26,460 (96.4)	32,253 (96.3)
2	251 (4.2)	951 (3.5)	1,202 (3.6)
3	8 (0.1)	40 (0.2)	48 (0.1)
Place of health checkups
Public facility	4,664 (77.1)	22,230 (81.0)	26,894 (80.3)
Medical institution	1,388 (22.9)	5,221 (19.0)	6,609 (19.7)
Prior prescriptions within one year before the health checkup
Antihypertensive	1,041 (17.2)	2,760 (10.1)	3,801 (11.4)
Antidiabetic	47 (0.8)	125 (0.5)	172 (0.5)
Antigout	110 (1.8)	277 (1.0)	387 (1.2)
Antidepressant	171 (2.8)	418 (1.5)	589 (1.8)
Number of months of physician visits in the previous year, median (IQR), month	4 (1-8)	3 (1-6)	3 (1-6)
Result of the health checkup in the previous fiscal year
Dyslipidemia	3,244 (53.6)	15,779 (57.5)	19,023 (56.8)
No dyslipidemia	1,220 (20.2)	5,730 (20.9)	6,950 (20.7)
No health checkup in the previous year	1,588 (26.2)	5,942 (21.7)	7,530 (22.5)

BMI, body mass index; IQR, interquartile range. The numbers are No. (%), unless otherwise specified.

The proportion of individuals who attended a follow-up visit was 18.1% (6,052/33,503). Table 2 shows the proportions according to dyslipidemia type and combination. The attendance proportions for individuals with high LDL-C, high TG, and low HDL-C levels were 17.9%, 20.7%, and 18.3%, respectively. The follow-up proportions of the 31,084 participants with high LDL-C levels were 15.7%, 18.6%, and 23.6% for LDL-C levels of 140-160 mg/dL, 160-180 mg/dL, and ≥180 mg/dL, respectively. For the 44 municipalities, the median (IQR) proportion of attendance was 18.4 (15.9%-19.9%). Stratified by population size, the attendance proportion was higher among urban residents (18.5% [5,553/30,083]) than rural residents (14.6% [499/3,420]). When we extended the follow-up period to 365 days, 26.1% (8,490/32,573) of individuals attended a follow-up visit within 365 days after the health checkup.

**Table 2.** Proportion of Attendance at a Follow-Up Physician Visit for Dyslipidemia, by Type and Combination of Dyslipidemia.

	Proportion, %
**Type of dyslipidemia**
LDL-C ≥ 140 mg/dL	17.9 (5,573/31,084)
LDL-C 140-160 mg/dL	15.7 (2,624/16,723)
LDL-C 160-180 mg/dL	18.6 (1,655/8,886)
LDL-C ≥ 180 mg/dL	23.6 (1,294/5,475)
TG ≥ 300 mg/dL	20.7 (574/2,779)
HDL-C ≤ 34 mg/dL	18.3 (172/938)
**Combination of dyslipidemia**
One type	18.0 (5,793/32,253)
High LDL-C only	17.9 (5,371/30,079)
High TG only	20.1 (333/1,654)
Low HDL-C only	17.1 (89/520)
Two types	20.9 (251/1,202)
High LDL-C and High TG	21.2 (176/832)
High LDL-C and Low HDL-C	14.4 (18/125)
High TG and Low HDL-C	23.3 (57/245)
High LDL-C, High TG, and Low HDL-C	16.7 (8/48)

LDL-C, low-density lipoprotein cholesterol; HDL-C, high-density lipoprotein cholesterol; TG, triglycerides.

As shown in Table 3, several characteristics showed statistically significant associations with attendance at a follow-up visit. In multivariable logistic regression analyses, younger age, male sex, occasional alcohol consumption, and health checkups at public facilities were associated with lower attendance. Individuals without symptoms or abnormal health checkup results (hypertension, hyperglycemia, liver dysfunction, proteinuria, and glycosuria) other than dyslipidemia were less likely to attend follow-up physician visits. Fewer dyslipidemias detected during the health checkup and fewer months of physician visits in the previous year were also associated with non-attendance. Individuals with prescriptions for hypertension, gout, or depression before the health checkup had higher odds of attendance, while those with antidiabetic prescriptions had lower odds of attendance. Individuals who did not receive a health checkup in the previous year, that is, those who received a health checkup for the first time in at least 2 years, were more likely to visit a physician for follow-up compared with those who received a health checkup in the previous fiscal year and had no dyslipidemia.

**Table 3.** Adjusted Odds Ratios for Attendance at a Follow-Up Physician Visit for Dyslipidemia.

n = 33,503	Unadjusted OR (95% CI)	Adjusted OR (95% CI)
**Predisposing factors**
Age group (years) (ref. 40-49 years old)
50-59	1.28*** (1.11-1.46)	1.24** (1.09-1.42)
60-69	1.62*** (1.46-1.79)	1.49*** (1.35-1.63)
70-74	1.86*** (1.62-2.14)	1.63*** (1.43-1.86)
Male sex	0.81*** (0.76-0.87)	0.79*** (0.74-0.85)
**Enabling factors**
Place of health checkups (ref. medical institution)
Public facility	0.79*** (0.73-0.85)	0.89* (0.83-0.96)
Number of months of physician visits in the previous year	1.08*** (1.07-1.09)	1.07*** (1.06-1.08)
Number of medical institutions in the municipality per 1,000 population	1.36 (0.94-1.97)	1.12 (0.77-1.63)
**Need factors**
Symptoms, yes	1.26*** (1.18-1.35)	1.17*** (1.10-1.25)
BMI (kg/m^2^) (ref. < 25)
25-30	1.04 (0.98-1.10)	0.95 (0.90-1.01)
≥30	1.14* (1.004-1.30)	0.96 (0.84-1.10)
Health checkup results
Hypertension (ref. no hypertension)	1.17*** (1.09-1.25)	1.08* (1.01-1.15)
Hyperglycemia (ref. no hyperglycemia)	2.08*** (1.85-2.34)	1.89*** (1.66-2.16)
Liver dysfunction (ref. no liver dysfunction)	1.34*** (1.19-1.50)	1.43*** (1.28-1.59)
Proteinuria (ref. no proteinuria)	2.10*** (1.77-2.49)	1.73*** (1.44-2.07)
Glycosuria (ref. no glycosuria)	1.99*** (1.64-2.41)	1.32** (1.07-1.61)
Prior prescriptions within one year before the health checkup
Antihypertensive (ref. no antihypertensive)	1.86*** (1.68-2.05)	1.22*** (1.09-1.35)
Antidiabetic (ref. no antidiabetic)	1.71** (1.15-2.54)	0.57* (0.36-0.90)
Antigout (ref. no antigout)	1.82*** (1.46-2.26)	1.35* (1.07-1.71)
Antidepressant (ref. no antidepressant)	1.88*** (1.59-2.22)	1.32* (1.10-1.58)
Result of the health checkup in the previous fiscal year (ref. no dyslipidemia)
Dyslipidemia	0.97 (0.91-1.02)	0.996 (0.94-1.05)
No health checkup in the previous fiscal year	1.26*** (1.15-1.37)	1.36*** (1.26-1.47)
Number of dyslipidemias at the health checkup	1.17** (1.05-1.30)	1.14* (1.01-1.29)
**Health behaviors**
Current smoking, yes	0.84** (0.76-0.93)	0.97 (0.88-1.08)
Frequency of alcohol consumption (ref. rarely)
Every day	0.85*** (0.79-0.92)	0.92 (0.85-1.002)
Occasionally	0.86*** (0.80-0.92)	0.91* (0.85-0.98)

BMI, body mass index; CI, confidence interval; OR, odds ratio. * P < 0.05, ** P < 0.01, ***P < 0.001

**Figure 3.**
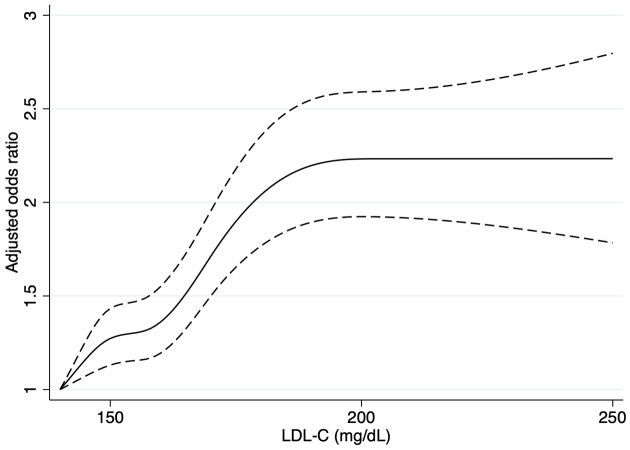
Association between LDL-C level and attendance at a follow-up physician visit for dyslipidemia. LDL-C, low-density lipoprotein cholesterol Note: Dashed lines indicate the 95% confidence intervals.

By restricting the analyses to 31,084 individuals with high LDL-C levels, the results were similar to those of the primary analysis, except for the lower odds of attendance among those who had dyslipidemia at the health checkup in the previous year compared with those who did not have dyslipidemia (Table 4). Regarding the association between the LDL-C level and non-attendance, individuals with LDL-C levels of 160-180 mg/dL (adjusted odds ratio [aOR], 1.30; 95% confidence interval [CI], 1.19-1.43) or ≥180 mg/dL (aOR, 1.82; 95% CI, 1.61-2.07) were more likely to attend the follow-up than those with LDL-C levels of 140-160 mg/dL. As shown in the restricted cubic spline (Figure 3), we observed a positive association between LDL-C levels and follow-up attendance, reaching a plateau at an LDL-C level of approximately 200 mg/dL.

**Table 4.** Adjusted Odds Ratios for Attendance at a Follow-Up Physician Visit for Dyslipidemia, among Those with High LDL-C.

n = 31,084	Unadjusted OR (95% CI)	Adjusted OR (95% CI)
**Predisposing factors**		
Age group (years) (ref. 40-49 years old)		
50-59	1.28*** (1.12-1.47)	1.22** (1.06-1.42)
60-69	1.62*** (1.45-1.82)	1.49*** (1.33-1.66)
70-74	1.88*** (1.62-2.17)	1.65*** (1.42-1.91)
Male sex	0.79*** (0.74-0.84)	0.81*** (0.76-0.86)
**Enabling factors**
Place of health checkups (ref. medical institution)
Public facility	0.79*** (0.73-0.86)	0.86*** (0.80-0.93)
Number of months of physician visits in the previous year	1.08*** (1.08-1.09)	1.07*** (1.06-1.08)
Number of medical institutions in the municipality per 1,000 population	1.39 (0.96-2.02)	1.12 (0.76-1.64)
**Need factors**
Symptoms, yes	1.26*** (1.18-1.35)	1.17*** (1.10-1.25)
BMI (kg/m^2^) (ref. < 25)
25-30	1.04 (0.97-1.11)	0.95 (0.88-1.01)
≥30	1.13 (0.99-1.29)	0.95 (0.84-1.08)
Health checkup results
Hypertension (ref. no hypertension)	1.19*** (1.11-1.27)	1.07* (1.001-1.15)
Hyperglycemia (ref. no hyperglycemia)	2.11*** (1.85-2.42)	1.91*** (1.63-2.24)
Liver dysfunction (ref. no liver dysfunction)	1.33*** (1.16-1.52)	1.38*** (1.21-1.57)
Proteinuria (ref. no proteinuria)	2.09*** (1.73-2.52)	1.73*** (1.41-2.11)
Glycosuria (ref. no glycosuria)	2.01*** (1.65-2.45)	1.34** (1.09-1.66)
Prior prescriptions within one year before the health checkup
Antihypertensive (ref. no antihypertensive)	1.83*** (1.66-2.02)	1.23*** (1.11-1.37)
Antidiabetic (ref. no antidiabetic)	1.81** (1.22-2.68)	0.61* (0.39-0.95)
Antigout (ref. no antigout)	1.90*** (1.48-2.45)	1.57** (1.19-2.06)
Antidepressant (ref. no antidepressant)	1.86*** (1.56-2.23)	1.25* (1.03-1.53)
Results of the health checkup in the previous fiscal year (ref. no dyslipidemia)
Dyslipidemia	0.98 (0.93-1.04)	0.86*** (0.79-0.92)
No health checkup in the previous fiscal year	1.27*** (1.16-1.39)	1.18*** (1.08-1.30)
Number of dyslipidemias at the health checkup	1.13* (1.001-1.28)	1.13 (0.99-1.30)
LDL-C level (mg/dL) (ref. 140-160 mg/dL)
160-180	1.23*** (1.13-1.34)	1.30*** (1.19-1.43)
≥180	1.66*** (1.49-1.86)	1.82*** (1.61-2.07)
**Health behaviors**
Current smoking, yes	0.83*** (0.75-0.92)	0.98 (0.88-1.09)
Frequency of alcohol consumption (ref. rarely)
Every day	0.82*** (0.75-0.89)	0.92 (0.84-1.01)
Occasionally	0.85*** (0.79-0.91)	0.91** (0.85-0.98)

BMI, body mass index; CI, confidence interval; LDL-C, low-density lipoprotein cholesterol; OR, odds ratio. * P < 0.05, ** P < 0.01, ***P < 0.001

## Discussion

This retrospective cohort study revealed that only 18% of individuals with dyslipidemia identified at the health checkup visited a physician for follow-up within 6 months. We also found several risk factors associated with non-attendance. To the best of our knowledge, this is the first study to investigate factors associated with non-attendance at follow-up physician visits for dyslipidemia identified at health checkups.

A previous study on company employees and their families reported that the proportion of individuals who attended a follow-up visit within 6 months after the health checkup was 15-21% for dyslipidemia ^(8)^. The current study on self-employed, unemployed, and part-time workers revealed that attendance at the follow-up for dyslipidemia (18.1%) was similarly low.

Concerning predisposing factors, younger age, male sex, and occasional drinking habits were associated with non-attendance. Younger age and male sex are reportedly risk factors for non-follow-up after the health checkup for hyperglycemia ^(9)^, and our results confirmed that these populations are at a high risk of missing follow-up physician visits for dyslipidemia. We could not determine the underlying mechanism; however, younger people may find it more difficult to devote their time to follow-up visits than older people.

In terms of enabling factors, individuals who received health checkups at public facilities (vs. medical institutions) were more likely to miss the follow-up visit. In addition, those who had fewer physician visits before the health checkup were more likely to miss the follow-up, consistent with a previous report on hyperglycemia ^(10)^. Meanwhile, the number of medical institutions in the municipality where the individual lived was not associated with non-attendance. A possible explanation is that those who underwent their checkups at public facilities or did not receive medical care for other diseases may have had fewer opportunities to consult with healthcare providers about the abnormal cholesterol levels identified at the checkup. Whether each individual has such an opportunity may be more important than whether there are medical institutions in the neighborhood.

With respect to the need factors, we found that participants without symptoms or other abnormal results at health checkups were less likely to visit a physician when dyslipidemia was identified. We also found that a lower LDL-C level was associated with non-attendance, consistent with previous studies on hyperglycemia and hypertension that reported lower HbA1c levels or blood pressure as predictors of non-attendance ^(9), (10), (12)^. They may not feel the need to visit a doctor unless the results are far out of the normal range. Also, we mentioned the frequency of physician visits as an enabling factor (access to regular sources), but it can also be a need factor reflecting the person’s illnesses.

Prescriptions for hypertension, gout, and depression were associated with higher attendance, whereas antidiabetic prescriptions were associated with lower attendance. The reason for this discrepancy is unclear, but it should be noted that not having undergone blood tests for cholesterol levels during the past year was required for entry into the study population. Accordingly, individuals with diabetes who had their cholesterol levels checked in the past year were excluded, even though it is common to test cholesterol levels in individuals with diabetes. Therefore, individuals with diabetes prescriptions in the current study may not represent the general characteristics of individuals with diabetes. For example, individuals with prescriptions for diabetes in this study may have included those who had diabetes but who were reluctant to undergo the routine blood test for their cholesterol levels.

Individuals who had dyslipidemia at a health checkup in the previous year were less likely to attend a follow-up visit, even after dyslipidemia was identified again the following year, which is consistent with a report on hyperglycemia ^(11)^. This phenomenon may reflect not only the lack of perceived needs of the individuals but also their health beliefs or values concerning their health.

In 2023, the Japanese government set a goal of reducing individuals with high LDL-C levels by 25% in the next 10 years ^(23)^. Our findings have several implications for healthcare providers and policymakers, which could be helpful when considering interventions to improve attendance at follow-up physician visits for those with abnormal cholesterol levels. First, sending reminders to those with a higher possibility of non-attendance at follow-ups may be efficient. Second, more tailored interventions could be considered. For example, for those who receive checkups at public facilities or do not usually visit doctors for other diseases, it may be effective to provide information on medical institutions where they can consult about dyslipidemia in their neighborhood because they may be less likely to have regular sources for consultation. As another example, communication about the increased risk of cardiovascular diseases caused by dyslipidemia may be important for those who do not have symptoms or other abnormal health checkup results.

This study had some limitations. First, our data lacked employment or economic status information, so we could not assess the association between these characteristics and non-attendance. Second, the generalizability of this study may be limited, although our data included more than 700,000 insured individuals covered by the National Health Insurance of the prefecture. Third, information bias is possible since some exposure variables (e.g., smoking and drinking status) were measured by the self-report questionnaires. Fourth, as we excluded those who became ineligible for the National Health Insurance within 180 days after the checkup, the selection bias could be possible. Fifth, some local municipalities or medical institutions may have used the thresholds different from the standard health checkup and health guidance program set by the Ministry of Health, Labour, and Welfare. However, we lacked information on the local rules, if any, and assumed the government’s standard threshold was widely used. Furthermore, we could not determine the origin of dyslipidemia (i.e., if it was primary or secondary to other diseases such as thyroid dysfunction) from our data. Finally, although we discussed several possible interventions to reduce non-attendance at follow-up visits, future intervention studies are required to assess their efficacy and whether such interventions can ultimately decrease the risk of cardiovascular events.

In conclusion, only 18% of individuals with dyslipidemia identified at health checkups attended follow-up physician visits within 6 months. We identified several risk factors for non-attendance. Interventions targeting these high-risk populations may be important.

## Article Information

### Conflicts of Interest

None

### Sources of Funding

This work was supported by a grant-in-aid from the Ministry of Health, Labour and Welfare Policy Research Grants, Japan [grant number 23AA2003]. The funder played no role in the conception, design, implementation, or reporting of the study.

### Acknowledgement

We are grateful to the staff members of Ibaraki Prefectural Government for their contributions to data acquisition. We thank Editage (www.editage.com) for its English language editing.

### Author Contributions

YT designed the study, analyzed the data, and drafted the manuscript. MI and TS contributed to conception, study design, interpretation, and revision of the manuscript. NK, TY, RI, AS, TW, FI, and NT substantially contributed to the interpretation of the data and critically revised the manuscript. All authors approved the final version.

### Approval by Institutional Review Board (IRB)

No.1845-1 (the University of Tsukuba)

## Supplement

Supplementary Table 1
