# Supplementary material for: Factors Associated with Non-attendance at a Follow-up Visit for Dyslipidemia Identified at Health Checkups: A Retrospective Cohort Study in a Japanese Prefecture
Source: JMA J. 2024 Oct 3;7(4):518–28. doi: 10.31662/jmaj.2024-0065 (PMC11543281; doi:10.31662/jmaj.2024-0065)
Supplement: Supplementary Table 1 [file 2433-3298-7-4-0518-s001.pdf]

Supplementary Table 1. Profile of municipalities in Ibaraki Prefecture, Japan (n = 44)

	Population[1] (2018)	National Health Insurance insured persons[2] (2018)	Number of medical institutions[3] (2018)
**City**			
Mito	273,243	61,246	264
Hitachi	182,391	34,846	125
Tsuchiura	143,024	35,309	116
Koga	144,480	37,513	87
Ishioka	76,062	19,415	58
Yuki	52,566	14,041	30
Ryugasaki	77,857	19,227	47
Shimotsuma	44,328	11,998	28
Joso	64,036	17,187	33
Hitachiota	53,020	12,921	25
Takahagi	29,232	6,735	22
Kitaibaraki	44,206	10,125	23
Kasama	76,969	19,869	43
Toride	108,049	27,089	63
Ushiku	85,255	20,091	60
Tsukuba	230,360	47,369	196
Hitachinaka	159,574	31,548	97
Kashima	68,057	18,005	42
Itako	28,641	8,139	13
Moriya	66,922	13,162	45
Hitachiomiya	42,860	11,491	26
Naka	55,230	13,288	36
Chikusei	106,013	27,896	86
Bando	55,057	16,947	27
Inashiki	42,123	12,148	19
Kasumigaura	42,413	10,808	23
Sakuragawa	42,990	12,170	21
Kamisu	95,354	23,682	42
Namegata	35,694	11,721	16
Hokota	49,742	19,658	21
Tsukubamirai	51,590	11,333	21
Omitama	51,747	13,746	25
**Town**			
Ibaraki	33,002	9,590	19
Oarai	17,203	5,154	11
Shirosato	20,029	5,537	9
Daigo	17,744	5,308	8
Ami	47,506	11,540	27
Kawachi	9,104	2,612	2
Yachiyo	22,722	7,670	6
Goka	8,740	2,485	3
Sakai	25,319	7,556	14
Tone	16,541	5,331	9
**Village**			
Tokai	38,405	7,044	18
Miho	15,687	4,061	5

Data source: 1. Basic Resident Register, Ministry of Internal Affairs and Communications. 2. National health insurance business situation, Ibaraki Prefectural Government. 3. Survey of Medical Institutions, Ministry of Health,Labour and Welfare.
